# Differential Neuroprotection of Selective Estrogen Receptor Agonists against Autonomic Dysfunction and Ischemic Cell Death in Permanent versus Reperfusion Injury

**DOI:** 10.1155/2011/976951

**Published:** 2011-04-28

**Authors:** Barry J. Connell, Tarek M. Saleh

**Affiliations:** Department of Biomedical Science, Atlantic Veterinary College, University of Prince Edward Island, Charlottetown, PE, Canada C1A 4P3

## Abstract

In the present study, we tested the hypothesis that selective activation of estrogen receptor subtypes (ER**α** and ER**β**) would be neuroprotective following ischemia and/or ischemia-reperfusion, as well as prevent the associated autonomic dysfunction. The selective ER**α** agonist, PPT, when administered 30 min prior to occlusion of the middle cerebral artery (pMCAO), resulted in a dose-dependent neuroprotection as measured 6 hours postpermanent MCAO, but not following 30 mins of MCAO followed by 5.5 hrs of reperfusion (I/R). In contrast, 30 min pretreatment with the selective ER**β** agonist, DPN, resulted in a dose-dependent neuroprotection following I/R, but was not protective following pMCAO. Both drugs prevented the ischemia-induced autonomic dysfunction as measured by a decrease in the baroreceptor reflex sensitivity (BRS). The data presented here suggest a differential role of each ER subtype in targeting the mechanisms of cell death that occur in ischemia versus reperfusion injury.

## 1. Introduction

The use of estrogen as a therapeutic agent in neurodegenerative diseases, in particular stroke, has been investigated over the last decade. Our laboratory, as well as several others, have demonstrated that estrogen significantly reduces ischemia-induced cell death following middle cerebral artery occlusion (MCAO) using multiple rodent models of permanent and transient ischemic stroke [1–5]. Unfortunately, this success against ischemic cell death observed in such preclinical studies following administration of estrogen to animals has not translated to successful clinical trial results in humans [6]. Although it is debatable if some of the clinical trial designs hold validity [7, 8], many researchers remain convinced that estrogen, or some intermediate molecular target(s) of estrogen, play a key role in neuroprotection following ischemic stroke, and ischemia-reperfusion (I/R) [9, 10]. As a result, many current investigations have examined the mechanism of estrogen-induced neuroprotection. 

Estrogen has been shown to result in both rapid and acute as well as long-term, chronic alterations in neuronal physiology, and this may be due to differential activation of estrogen receptor (ER) subtypes, one of which may reside on the cell membrane. Two main estrogen receptor subtypes have been identified, ER*α*, ER*β*, and the putative membrane-bound receptor GPR30 [11]. To date, the issue of which ER subtype plays a predominant role in estrogen-mediated neuroprotection has not been resolved. These studies suggest that both ER*α* and ER*β* receptor subtypes are expressed in the cerebral cortex in adult rats [12, 13]. Several laboratories have proposed that the ER*α* receptor subtype is more important in estrogen-mediated neuroprotection in animal models of cerebral ischemia [14–16]. Merchenthaler and colleagues [14] determined that ER*α* was responsible for mediating the neuroprotective actions of estrogen following permanent MCAO. These authors also demonstrated that the penumbra contained a large number of immunoreactive and mRNA-expressing ER*α* positive cells, and this was only observed on the ipsilateral, but not the contralateral side. Further support for the neuroprotective effects of ER*α* comes from Dubal and colleagues [15] who reported that deletion of ER*β*, using ER*β* knockout mice, had no effect on the estrogen-induced protection following ischemia, whereas the protective actions of estrogen were completely abolished in ER*α* knockout mice. In a more recent study, Dubal and colleagues [16] investigated the temporal expression of ER*α* following permanent MCAO and reported a significant induction of ER*α* mRNA in the ischemic region early in the development of the infarct. In contrast, Farr and colleagues [17] did not observe a neuroprotective effect of ER*β* activation in a rat model of permanent focal ischemia. However, activation of ER*β* has been shown to promote neuroprotection against glutamate excitotoxicity in hippocampal neurons [18]. 

Our laboratory has previously demonstrated that systemic estrogen administration significantly enhanced autonomic function as measured by an increase in the sensitivity of the baroreceptor reflex (BRS) in both male and female rats [19–22]. Further, we have demonstrated that estrogen acts centrally to improve sympathovagal balance by decreasing sympathetic tone and increasing parasympathetic tone [23–25]. The BRS is depressed following the onset of several cardiovascular pathologies [26, 27] including stroke and the administration of systemic estrogen has been shown to decrease both the stroke-induced depression in the BRS and stroke-induce ischemia [4].

The aim of these experiments is to determine if a dichotomy exists between the ability of ER*α* or ER*β* receptor subtypes to mediate neuroprotection in both permanent and transient models of cerebral ischemia in a single study. Also, we set out to determine if selective ER receptor agonists also mediate protection against the stroke-induced autonomic dysfunction as measured by the ischemia-induced depression in the sensitivity of the BRS.

## 2. Methods

All experiments were carried out in accordance with the guidelines of the Canadian Council on Animal Care and were approved by the University of Prince Edward Island Animal Care Committee.

### 2.1. General Surgical Procedures

All experiments were conducted on male Sprague-Dawley rats (90 rats; 250–350 g; Charles Rivers; Montreal, PQ, Canada). For all animals, food and tap water were available* ad libitum*. Rats were anaesthetized with sodium thiobutabarbital (Inactin; Sigma-Aldridge; St. Louis, MO, USA; 100 mg/kg; ip) which provided a stable plane of anesthesia for the full duration of the experimental time periods (no animals required anesthetic supplementation). To monitor blood pressure and heart rate, a polyethylene catheter (PE-50; Clay Adams, Parsippany, NJ, USA) was inserted into the right femoral artery. For intravenous administration of drugs, a polyethylene catheter (PE-10; Clay Adams, Parsippany, NJ, USA) was inserted into the right femoral vein. Arterial blood pressure was measured with a pressure transducer (Gould P23 ID, Cleveland, OH) connected to a Gould model 2200S polygraph. Heart rate was determined from the pulse pressure using a Gould tachograph (Biotach). These parameters were displayed and analyzed using PolyviewPro/32 data acquisition and analysis software (Grass; Warwick, RI, USA). An endotracheal tube was inserted to facilitate spontaneous breathing on room air. Body temperature was monitored and maintained at 37 ± 1°C using a Physitemp feedback system (Physitemp Instruments; Clifton, NJ, USA). 

4,4′,4′′-(4-propyl-[1H]-pyrazole-1,3,5-triyl)trisphenol (PPT; Tocris Bioscience) and 2,3-bis(4-hydroxyphenyl)-propionitrile (DPN; Tocris Bioscience) were used to selectively activate estrogen alpha (ER*α*) and beta (ER*β*) receptors, respectively.

### 2.2. Middle Cerebral Artery Occlusions (MCAO)

Our lab had previously published the detailed methodology for transient occlusion of the middle cerebral artery [1]. Briefly, animals were placed in a David Kopf stereotaxic frame (Tujunga, CA, USA) and the right middle cerebral artery (MCA) approached through a rostral-caudal incision of the skin and frontalis muscle at the approximate level of bregma. Blood flow through the MCA was impeded by the placement of surgical suture behind the MCA at 3 designated positions along the exposed vessel. The ends of the sutures were positioned so that the middle of the each suture applied pressure to the MCA and impeded blood flow. This 3-point placement of surgical sutures produced a highly reproducible and consistent focal ischemic lesion restricted to the ipsilateral cerebral cortex. To facilitate removal of the sutures at the end of the occlusion period (30 minutes), a few drops of warm physiological saline (37°C) was first applied to the areas where the MCA was in contact with the sutures. Blood was allowed to reperfuse the area for an additional 5.5 hours (I/R). At the end of the 6 hour occlusion (pMCAO) or I/R, all animals were perfused transcardially with phosphate buffered saline (PBS; 0.1 M; 200 mls), the brains removed and sliced into 1 mm coronal sections using a rat brain matrix (Harvard Apparatus; Holliston, MA, USA).

### 2.3. Cardiac Baroreflex Testing

To determine the effect of estrogenic agonists and MCAO or I/R on the reflex bradycardia following baroreceptor activation, the baroreceptor reflex was evoked using a bolus intravenous injection of the *α*-adrenergic receptor agonist phenylephrine-hydrochloride (Sigma-Aldridge; 0.1 mL; 2.5 *μ*g/mL; iv). The ratio of the peak change in the magnitude of the reflex bradycardia to the magnitude of the phenylephrine-induced pressor response (ΔHR/ΔMAP) was used as a measure of BRS. The BRS was tested using PE injected at 10 min intervals prior to drug injection and MCAO and then at regular intervals following occlusion and/or reperfusion.

### 2.4. Effect of PPT and DPN on Permanent or Reperfusion-Induced Infarct Volume

In the first experiment, to examine the effect of estrogen receptor alpha activation on both permanent ischemia and ischemia-reperfusion-induced cell death, injections of PPT (0.01, 0.05, 0.1, or 1.0 mg/kg; 1 mL/kg; iv; *n* = 4/group) or dimethyl sulfoxide (DMSO; 50%; 1 mL/kg; iv; *n* = 4) were made 30 minutes (−30 minutes) prior to MCAO. In the first group, the sutures were left in place for 30 minutes, followed by 5.5 hours of reperfusion (I/R). In the second group, the sutures were left in place for 6 hours (pMCAO). The cardiac BRS was tested at 10 min intervals prior to and during pMCAO and I/R. In the second experiment, to examine the effect of estrogen receptor beta activation on both permanent ischemia and ischemia-reperfusion-induced cell death, injections of DPN (0.01, 0.1, or 1.0 mg/kg; 1 mL/kg; iv; *n* = 4 to 6/group) or dimethyl sulfoxide (DMSO; 7.5%; 1 mL/kg; iv; *n* = 6) were made 30 minutes (−30 minutes) prior to pMCAO or I/R. The cardiac BRS was tested as described above.

### 2.5. Histological Procedures

Sections were incubated in a 2% solution of 2,3,5-triphenol tetrazolium chloride (TTC; Sigma-Aldrich; St. Louis; MO, USA) for 5 minutes. Infarct volumes were calculated with the use of scanned digital images of each brain section. Infarct areas were calculated using a computer-assisted imaging system (Scion Corporation; Frederick, MD, USA). The infarct areas for each side for each individual section were averaged and multiplied by the width of each section (1 mm) to give the infarct volume for each section. The sum total of all the individual infarct volumes provided the infarct volume for each rat.

### 2.6. Statistical Analysis

Data were analyzed using a statistical software package (SigmaStat and SigmaPlot; Jandel Scientific, Tujunga, CA, USA). All data are presented as a mean ± standard error of the mean (S.E.M.). Differences were considered statistically significant if *P* ≤ .05 by an analysis of variance (ANOVA) followed by a Bonferroni post-hoc analysis or repeated measures (BRS). When only two groups were being compared the Student's *t*-test was used.

## 3. Results

### 3.1. The Effect of Administration of PPT on Infarct Volume Following Permanent MCAO and I/R

**Figure 1:**
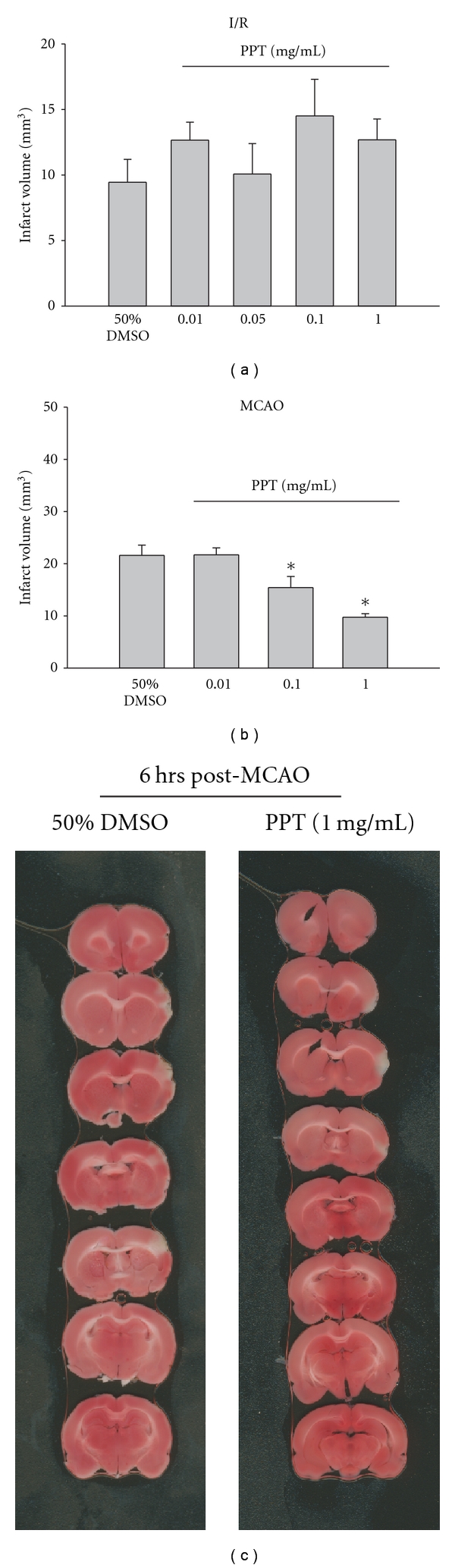
Effect of pretreatment with either 50% DMSO or PPT (i.v.; 30 minutes) on infarct volume (mm^3^) calculated from TTC-stained 1 mm thick coronal sections throughout the extent of the infarct following I/R (a) and MCAO (b). Each bar represents the mean ± S.E.M (*n* = 4–6/group), and *indicates significance (*P*  ≤  .05) from the DMSO control group. (c) Representative photomicrographs of TTC stained, 1 mm thick coronal slices illustrating the extent of the infarct within the prefrontal cortex following 30 minutes pretreatment (i.v.) with either DMSO or PPT (1 mg/kg) at 6 hours post-MCAO.

The following experiment was designed to determine the effect of the estrogen receptor alpha (ER*α*) agonist, PPT, on pMCAO and I/R. 30 min pretreatment with PPT followed by I/R did not produce significant neuroprotection compared to vehicle (*P* ≥ .05; Figure 1(a)). In contrast, pretreatment with PPT prior to 6 hours of pMCAO resulted in a dose-dependent neuroprotection, with doses of 0.1 and 1.0 mg/kg resulting in a significant decrease in infarct volume compared to the administration of vehicle (*P* ≤ .05; Figures 1(b) and 1(c)).

### 3.2. The Effect of Administration of DPN on Infarct Volume Following Permanent MCAO and I/R

**Figure 2:**
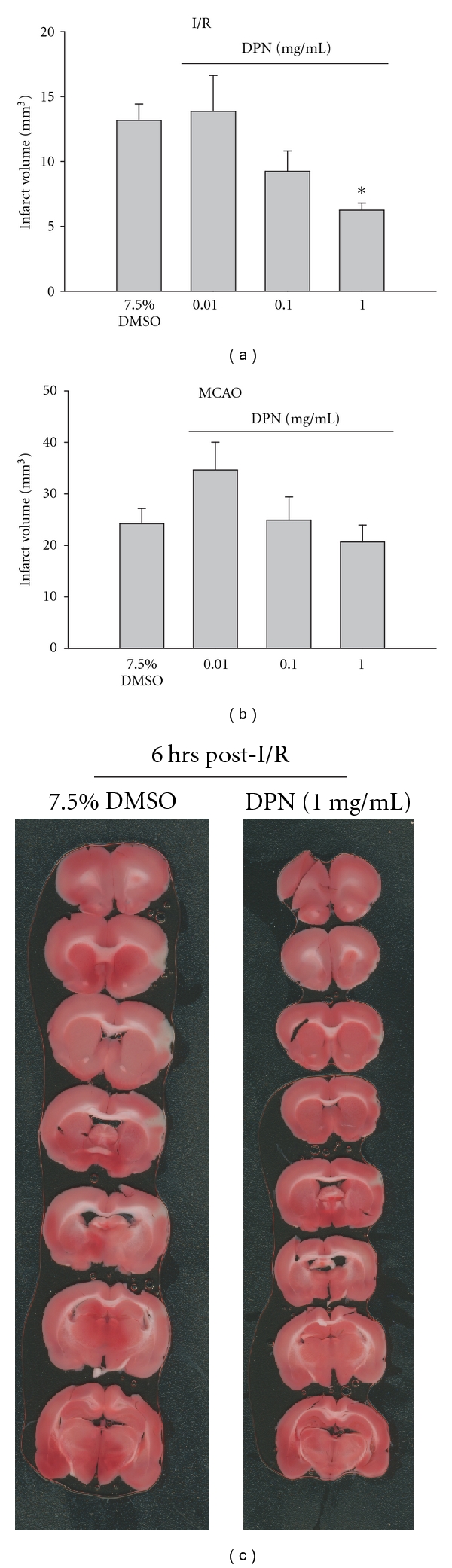
Effect of pretreatment with either 7.5% DMSO or DPN (i.v.; 30 minutes) on infarct volume (mm^3^) calculated from TTC-stained 1 mm thick coronal sections throughout the extent of the infarct following I/R (a) and MCAO (b). Each bar represents the mean ± S.E.M (*n* = 4–6/group), and *indicates significance (*P*  ≤  .05) from the DMSO control group. (c) Representative photomicrographs of TTC stained, 1 mm thick coronal slices illustrating the extent of the infarct within the prefrontal cortex following 30 minutes pretreatment (i.v.) with either DMSO or DPN (1 mg/kg) at 6 hours post-I/R.

The following experiment was designed to determine the effect of the estrogen receptor beta (ER*β*) agonist, DPN, on pMCAO-induced ischemia and I/R. DPN (1.0 mg/kg) produced significant neuroprotection compared to vehicle when administered 30 minutes prior to I/R (*P* ≤ .05; Figures 2(a) and 2(c)). In contrast, DPN pretreatment prior to 6 hours of pMCAO did not result in significant neuroprotection compared to the administration of vehicle (*P* ≥ .05; Figure 2(b)).

### 3.3. The Effect of Preadministration of PPT on Cardiovascular and Autonomic Parameters

**Figure 3:**
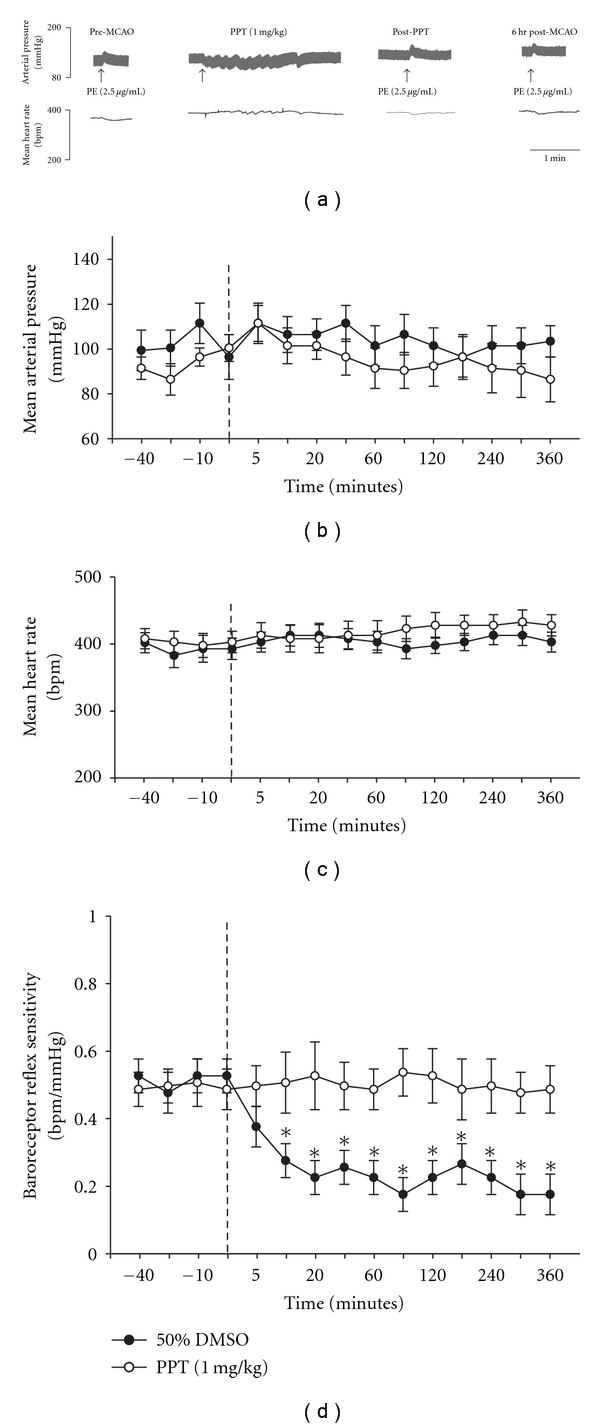
Cardiovascular responses to PPT (1 mg/kg) or 50% DMSO pretreatment (i.v.; 30 minutes) prior to MCAO. (a) Representative physiograph tracings of changes in arterial pressure and heart rate to phenylephrine injection (PE; 2.5 *μ*g/mL). Graphs represent average changes in mean arterial pressure (MAP; (b)), heart rate (HR; (c)), and baroreceptor reflex sensitivity (BRS; (d)). The dashed lines represents the times at which the MCA was occluded. Each data point represents the mean ± S.E.M (*n* = 4–6/group), and *indicates significance (*P*  ≤  .05) from baseline values.

The following experiment was designed to determine the effect of preadministration of a neuroprotective dose of PPT (1.0 mg/kg) on blood pressure, heart rate, and BRS before and during 6 hours of pMCAO. Preadministration of PPT or vehicle (50% DMSO) did not significantly alter mean arterial blood pressure or mean heart rate prior to, during, or following occlusion (*P* ≥ .05 compared to pre-pMCAO values, Figures 3(a), 3(b) and 3(c)). In the vehicle treated group, there was a significant decrease in BRS from 10 mins post-pMCAO to the end of the experiment (6 hours post-pMCAO; *P* ≤ .05 for each time point; Figure 3(d)). Administration of PPT completely blocked the depression in BRS at all time intervals measured post-pMCAO (*P* ≥ .05; for each time point compared to baseline and vehicle at the same time point; Figure 3(d)).

### 3.4. The Effect of Preadministration of DPN on Cardiovascular Parameters

**Figure 4:**
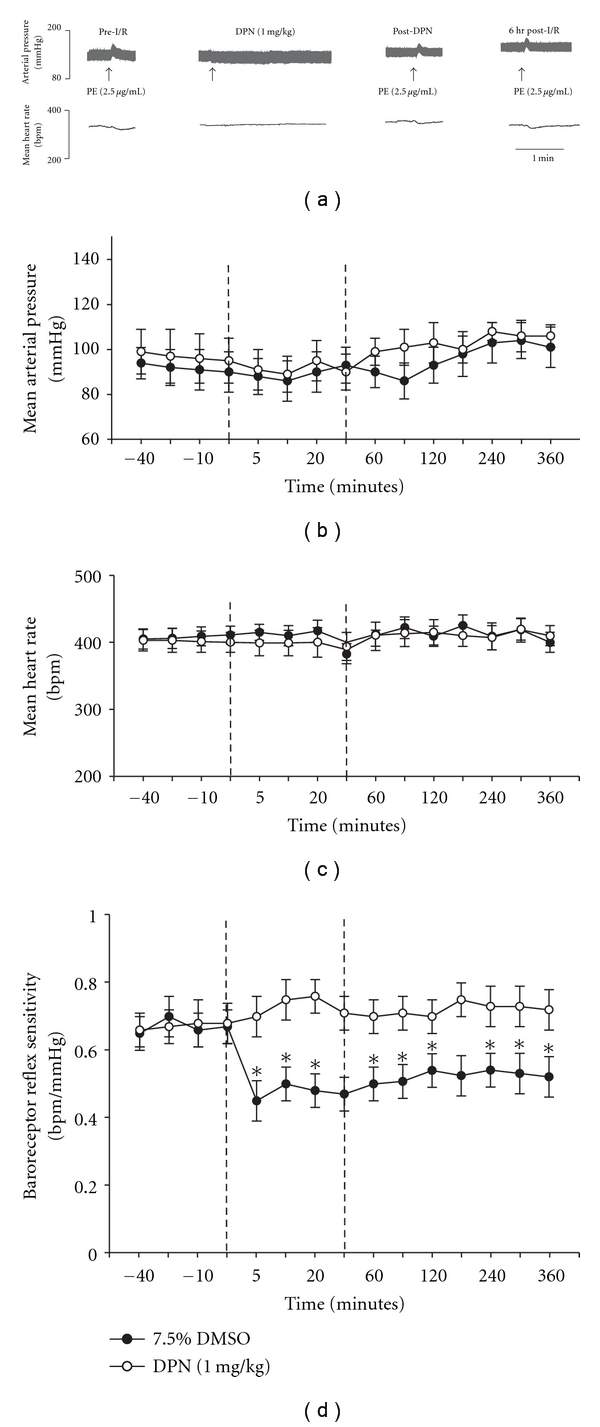
Cardiovascular responses to DPN (1 mg/kg) or 7.5% DMSO pretreatment (i.v.; 30 minutes) prior to I/R. (a) Representative physiograph tracings of changes in arterial pressure and heart rate to phenylephrine injection (PE; 2.5 *μ*g/mL). Graphs represent average changes in mean arterial pressure (MAP; (b)), heart rate (HR; (c)), and baroreceptor reflex sensitivity (BRS; (d)). The first dashed lines represents the times at which the MCA was occluded and the second line indicates when blood flow was returned (reperfusion). Each data point represents the mean ± S.E.M (*n* = 4–6/group), and *indicates significance (*P* ≤  .05) from baseline values.

The following experiment was designed to determine the effect of preadministration of a neuroprotective dose of DPN (1.0 mg/kg) on blood pressure, heart rate, and BRS before and following 30 minutes of MCA occlusion followed by 5.5 hours of reperfusion (I/R). Preadministration of DPN or vehicle (7.5% DMSO) did not significantly alter mean arterial blood pressure or mean heart rate prior to, during, or following occlusion (*P* ≥ .05; Figures 4(a), 4(b), and 4(c)). In the vehicle-treated group, BRS decreased significantly both during MCAO (30 mins) and immediately following reperfusion ()I/R) and continued depressed for the remained of the experiment (*P* ≤ .05; for each time point; Figure 4(d)). Administration of DPN 30 mins prior to MCAO completely blocked this depression of the BRS during both the 30 mins of ischemia as well as the 5.5 hours of reperfusion. The BRS remained not significantly different from baseline values measured prior to DPN administration for the full 5.5 hours post-MCAO (*P* ≥ .05; for each time point; Figure 4(d)).

## 4. Discussion

The results of the present investigation suggest that the selective activation of the estrogen receptor subtypes, ER*α* or ER*β*, differentially provide neuroprotection within the cerebral cortex against occlusive and reperfusion injury, respectively, as seen following permanent or transient MCAO. Our observations suggest that within the initial 6 hours following an ischemic insult, ER*α* protects against pMCAO-induced cell death only, while ER*β* provides neuroprotection against I/R-induced cell death only. Further, activation of either ER*α* or ER*β* can prevent the depression in the BRS observed following either pMCAO or I/R. These results suggest that estrogen receptor subtypes play different roles in protecting against the cell death associated with ischemia versus reperfusion injury; however, either receptor can participate in the prevention of the stroke-induced autonomic dysfunction. 

The exact events that lead from ischemia to cell death are not fully understood. However, convincing evidence supports the suggestion that excitotoxicity follows the hypoxic and hypoglycemic conditions following stroke [28]. Estrogen has been shown to protect against ischemia-induced excitotoxic injury in various *in vivo* and *in vitro* models of stroke [3]. The exact mechanisms of estrogen-induced neuroprotection are still being studied by several laboratories, including the role of estrogen receptor subtypes. Estrogen has been demonstrated to be an effective neuroprotectant in similar models of both permanent and transient MCAO [1, 4]. The data presented here now demonstrate that activation of the ER*α* subtype may have been responsible for the neuroprotective effect of estrogen seen in the permanent MCAO model, whereas activation of ER*β* may play a more prominent role in neuroprotection against reperfusion injury following transient ischemia. 

A prominent role for the ER*α* receptor subtype in estrogen-mediated neuroprotection in animal models of cerebral ischemia has been well documented [14–16]. Our current results are consistent with this suggestion as the prior administration of the ER*α* agonist, PPT, provided significant neuroprotection in our model of pMCAO, while the administration of the same dose ranges of the ER*β* agonist, DPN, was not able to provide significant neuroprotection against pMCAO-induced ischemia. Further evidence for this suggestion was provided by Dubal and colleagues [15] who reported that deletion of ER*β*, using ER*β* knockout mice, had no effect on the estrogen-induced protection following ischemia, whereas the protective actions of estrogen were completely abolished in ER*α* knockout mice. This finding was later supported by Dubal and colleagues [16] following an investigation of the temporal expression of ER*α* following pMCAO and reported a significant induction of ER*α* mRNA in the ischemic region within 4 hours of the development of the infarct. In a study conducted by Merchenthaler and colleagues [14], they demonstrated that the penumbra contained a large number of ER*α* mRNA-expressing and ER*α*-immunoreactive cells and this was only observed on the ipsilateral, but not the contralateral side. Finally, Sampei and colleagues [29] excluded a role for ER*α* involvement in the estrogen-mediated neuroprotection observed in their transient model of ischemia. These studies support our observation that ER*α* is responsible for mediating the neuroprotective actions of estrogen following pMCAO. 

Although a great deal of evidence supports the role of ER*α* in estrogen-induced neuroprotection in animal models of ischemia, ER*β* has also been shown to mediate the beneficial effects of estrogen in the brain. Wang and colleagues [30] demonstrated the importance of ER*β* in neuronal survival as developmental abnormalities occurred in ER*β* knockout mice. In addition, activation of ER*β* using DPN was shown to promote neuroprotection against glutamate excitotoxicity in hippocampal neurons in rats [18] and in a mouse model of global ischemia [31]. We now report that selective activation of ER*β* resulted in neuroprotection in a transient MCAO model but not in a permanent model of MCAO in rats. These results are in agreement with those published by Farr and colleagues [17] who also did not observe a neuroprotective effect of ER*β* activation in a rat model of permanent focal ischemia. 

During an occlusive episode, hypoxia and hypoglycemia produce an ischemic core where failure of ATP-dependent pumps lead to disruption of ionic equilibrium, calcium homeostasis, excitotoxicity, and eventual cell death [32]. Reintroduction of blood flow to underperfused neurons (e.g., with thrombolytic therapy) can result in reperfusion-injury in neurons that are in anoxic compensation and lead to the generation of toxic levels of oxidative free radicals [33, 34] resulting in lipid peroxidation, protein synthesis arrest, and cell death [34]. Estrogen has been shown to have antioxidant capabilities [35], and this may suggest a mechanism by which estrogen mediates neuroprotection against reperfusion-injury. It is possible that administration of the ER*β* agonist may act within the penumbra and ischemic area as an antioxidant, or by increasing the cells antioxidant capabilities, and this ability may or may not be related to activation of the estrogen receptor subtype. Both an investigation of the molecular mechanisms mediating the ER*β*-induced neuroprotection as well as development of selective ER*β* antagonists will be beneficial in answering this question. 

Clinically elevated sympathetic tone (sympathoexcitation), depressed parasympathetic tone, and abnormal electrocardiograms have been observed within 1 to 2 hours following thrombolytic or hemorrhagic stroke involving the MCA [36, 37], and such autonomic dysfunction increases the risk of sudden cardiac death [36, 37]. Arrhythmogenesis and sudden cardiac death which can occur following pMCAO in humans is associated with a depressed BRS [27]. Sympathoexcitation [38] and a depressed BRS can be mimicked in rat models of pMCAO [4]. Previous work from our laboratory have demonstrated that the prior administration of estrogen completely blocked the pMCAO-induced increase in sympathetic tone and decrease in BRS [4]. Further, we also demonstrated that this estrogen-mediated protection against the pMCAO-induced autonomic dysfunction was estrogen receptor dependent, as the effect was blocked by the prior or concomitant administration of the selective estrogen receptor antagonist, ICI-182,780 [4]. We have demonstrated in this study that prior administration of selective estrogen receptor agonist for either ER*α* (PPT) or ER*β* (DPN) subtypes produced functional protection by blocking the depression in the BRS observed following pMCAO and I/R. The use of selective ER*α* and ER*β* receptor antagonists will allow us to confirm if the functional protection observed was mediated via the selective activation of ER*α* or ER*β* receptor subtypes or through PPT and/or DPN-induced nonreceptor-mediated effects. Additional studies in our laboratory will focus on the potential mechanism of this estrogen receptor subtype activation in preventing the attenuated BRS. Possible options include an action directly on sympathetic or parasympathetic preganglionic neurons in the brain and/or spinal cord, an action on systemic arterial vasculature or even a direct action on cardiac myocytes which have been shown to express both estrogen receptor subtypes. 

In the present study, we observed that the most effective dose of the ER*α* agonist PPT, and the ER*β* agonist DPN, on both neuroprotection and autonomic protection following pMCAO or I/R was 1.0 mg/kg, which is 100 times greater than the optimal dose of 17*β*-estradiol (0.01 mg/kg) observed in similar studies in our laboratory [4, 5]. The greater dose requirement of these selective agonists compared to estradiol indicates that the activation of both receptor subtypes at the same time by estradiol may produce a synergistic effect on the neuroprotective mechanisms activated or that estradiol is a more potent antioxidant/free radical scavenger (a nonreceptor-mediated effect) that either selective agonist. 

In conclusion, our results suggest that each estrogen receptor subtype selectively and differentially protect against permanent or reperfusion injury following MCAO in rats. Also, we determined that both selective ER*α* and ER*β* agonists mediate functional protection against stroke-induced autonomic dysfunction as measured by the depression in the BRS. These results may provide insight into the development of targeted therapeutic strategies against ischemia and I/R-induced cell death and the subsequent cardiovascular consequences following stroke.
